# Supplementary material for: The interactome of intact mitochondria by cross-linking mass spectrometry provides evidence for coexisting respiratory supercomplexes
Source: Mol Cell Proteomics. 2017 Dec 8;17(2):216–32. doi: 10.1074/mcp.RA117.000470 (PMC5795388; doi:10.1074/mcp.RA117.000470)
Supplement: Supplemental Data [file supp_RA117.000470_133922_0_supp_23978_fzffwf.zip › spectra_annotation/mito_spectra_annotation/12-1-4-1-13-1.pdf]

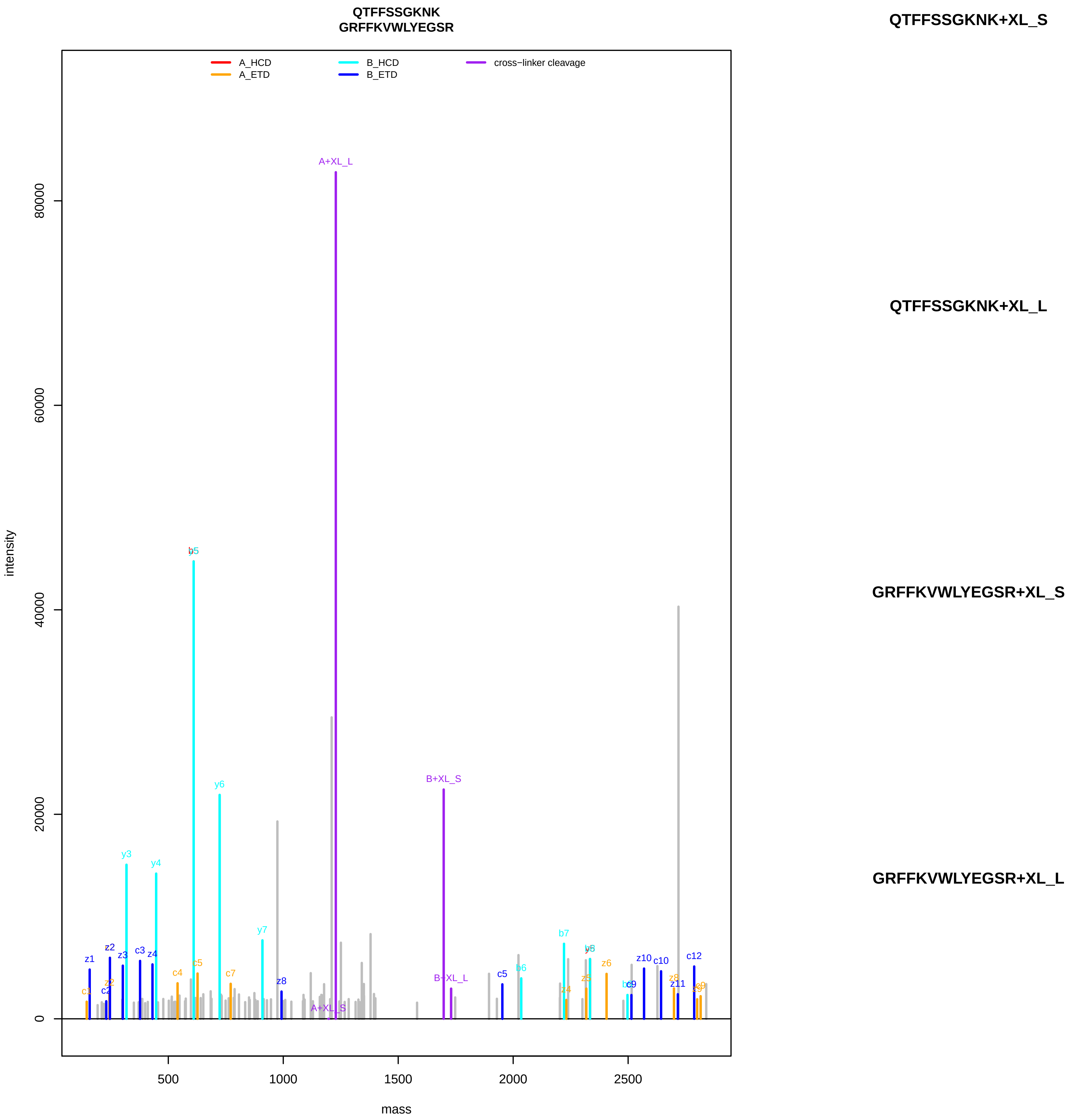

QTFFSSGKNK
GRFFKVWLYEGSR
A_HCD
A_ETD
B_HCD
B_ETD
cross−linker cleavage
A+XL_L
B+XL_S
B+XL_L
A+XL_S
b5
y3
y4
y6
y7
b6
b7
c1
c2
c3
c4
c5
c7
c5
c9
c10
c12
z1
z2
z3
z4
z6
z8
z10
z11
intensity
0
20000
40000
60000
80000
mass
500
1000
1500
2000
2500
QTFFSSGKNK+XL_S
QTFFSSGKNK+XL_L
GRFFKVWLYEGSR+XL_S
GRFFKVWLYEGSR+XL_L
